# RNase T2 in Inflammation and Cancer: Immunological and Biological Views

**DOI:** 10.3389/fimmu.2020.01554

**Published:** 2020-08-13

**Authors:** Lei Wu, Yanquan Xu, Huakan Zhao, Yongsheng Li

**Affiliations:** ^1^Department of Medical Oncology, Chongqing University Cancer Hospital, Chongqing, China; ^2^Clinical Medicine Research Center, Xinqiao Hospital, Army Medical University, Chongqing, China

**Keywords:** RNase T2, immunity, inflammation, cancer, toll-like receptors

## Abstract

The RNase T2 family consists of evolutionarily conserved endonucleases that express in many different species, including animals, plants, protozoans, bacteria, and viruses. The main biological roles of these ribonucleases are cleaving or degrading RNA substrates. They preferentially cleave single-stranded RNA molecules between purine and uridine residues to generate two nucleotide fragments with 2'3'-cyclic phosphate adenosine/guanosine terminus and uridine residue, respectively. Accumulating studies have revealed that RNase T2 is critical for the pathophysiology of inflammation and cancer. In this review, we introduce the distribution, structure, and functions of RNase T2, its differential roles in inflammation and cancer, and the perspective for its research and related applications in medicine.

## Introduction

**Figure 1:**
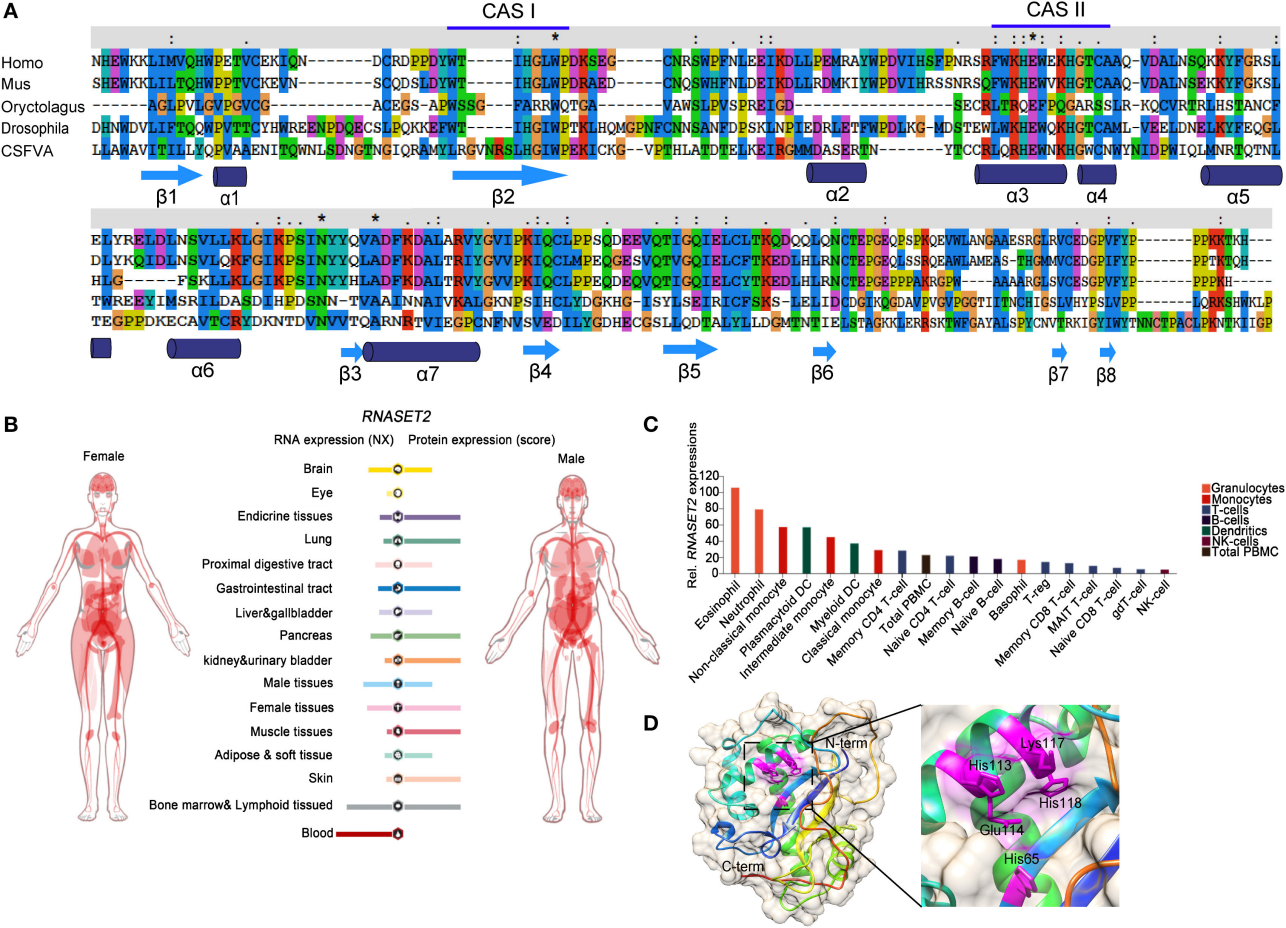
The evolutionary conservation structure of RNase T2 and distribution of RNASET2 in human tissues and immune cells. **(A)** Amino acid sequence of RNases T2 from human, mouse, rabbit, fruit fly, and classical swine fever virus showing the wide evolutionary conservation of these enzymes. CASI and CASII are indicated in the amino acid sequence, highly conserved AA are indicated as *. **(B)** Expressions of *RNASET2* in human tissues were analyzed in the Human Protein Atlas database, and the resulting transcript expression values, denoted normalized expression (NX), were calculated for each gene in every sample. **(C)** Expressions of *RNASET2* in 18 types of human blood cells and total peripheral blood mononuclear cells (PBMC) were analyzed in the Blood Atlas database. **(D)** The 3D structure of human RNase T2; the key catalytic residues His 65, His 113, Glu 114, Lys 117, and His 118 are shown in magenta.

Ribonucleases (RNases) are RNA-processing or -degrading enzymes that hydrolyze phosphodiester bonds within RNA molecules (1). According to their base specificity, structure, function, and optimal pH, RNases can be classified into the T1, A, and T2 families (2). All RNase T2 family members exhibit a conserved α/β core structure. Two conserved active site (CAS) motifs, I and II, are critical for the catalytic activity (3). RNA cleavage is promoted by one to three histidine residues that are located in CAS I and II. Mutations in these histidine residues lead to the inactivation of RNase T2 both *in vivo* and *in vitro* (Figure 1A). The RNase T2 family are widely distributed in living organisms and highly conserved from viruses to mammals (1). In human, RNase T2 is the only identified member of the RNase T2 family (4). This enzyme is detected in all tissues, especially in embryonic tissues and immune cells (https://www.proteinatlas.org/) (Figures 1B,C). The full-length human RNase T2 has 256 amino acids (AA) and a predicted size of 29 kD (Table 1). Human RNase T2 shows a typical structure, containing seven α-helices and eight β-strands. The catalytic site includes residues His 65, His 113, Glu 114, Lys 117, and His 118 (4). Most of these residues are located in α3 and α4 regions (Figure 1D).

**Table 1:** Comparison of the characteristics of human RNases.

**Name**	**Amino acid number**	**Protein molecular weight (kD)**	**Localization**	**Substrate**	**RNA sensor**	**Cleavage site**	**Fragment**	**References**
RNase T2	256	29	Lysosome, mitochondria, endoplasmic reticulum, vacuole, secreted	Bacterial, protozoan ssRNA, dsRNA	TLR8	Before uridine	Oligonucleotides with cyclic 2′,3′-phosphate termini and uridine	(3, 26, 32, 33)
RNase L	741	83	Mitochondria, cytosol	Viral and self-cellular dsRNA, ssRNA	TLR3, NLRP3, RIG-I	UA, UU	Small structured RNAs with 5′-OH and 3′-monophosphoryl, cyclic 2',3'-phosphate	(34–36)
IRE-1	977	109	Endoplasmic reticulum membrane	Endoplasmic reticulum-localized mRNA	RLR	XBP1-like consensus site	mRNA fragments	(37, 38)
RNase 1	156	17	Secreted	Extracellular self RNA	Unknown	Unknown	Unknown	(9, 39, 40)
RNase 2	161	18	Lysosome, cytoplasmic granule, secreted	Bacterial, protozoan, viral ssRNA, dsRNA	TLR8	After uridine	Oligonucleotides with cyclic 2′,3′-phosphate termini and uridine	(9, 26, 39)
RNase 3	160	18	Secreted	Bacterial, viral ssRNA	Unknown	Unknown	Unknown	(41, 42)
RNase 4	147	16	Secreted	viral ssRNA	Unknown	3′ side of uridine	Unknown	(43, 44)
RNase 5	147	16	Nucleus, secreted	Bacterial, fungi, viral, tumor cells snRNA, tRNA, rRNA	Unknown	UA, CA	Oligonucleotides (3′ tRFs)	(45–48)
RNase 6	150	17	Lysosome, cytoplasmic granule, secreted	Bacterial, viral ssRNA	Unknown	Unknown	Unknown	(9, 49)
RNase 7	156	17	Secreted	Bacterial, fungi, ssRNA	Unknown	Unknown	Unknown	(49–51)
RNase 8	154	17	Secreted	Bacterial ssRNA	Unknown	Unknown	Unknown	(52)

RNase T2 includes both intracellular and secretory types (5). The intracellular RNase T2 is mainly localized in lysosomes, mitochondria, vacuoles, and other organelles. The intracellular distribution pattern suggests that RNase T2 may be involved in degrading exogenous or endogenous RNAs in lysosome and regulating mitochondrial RNA metabolism (6–8). The secretory RNase T2 is proposed to have immunomodulatory and antimicrobial properties involved in host defenses (9). The expression and secretion of RNase T2 can be induced in response to a variety of tissue injury stimuli or oxidative stress (10, 11). Following tissue damage, RNase T2 is secreted and participates in resistance against RNA viruses or functions as an alarm signaling molecule to regulate the host immune response and contributes to tissue remodeling and repair (12, 13).

Accumulating studies have revealed that human RNase T2 participates in many biological processes such as angiogenesis, biogenesis of ribosomes, apoptosis, proliferation, and immune regulation (14, 15). Altered expression of RNase T2 is involved in various diseases, including autoimmune diseases and cancers (5, 16–19). For instance, RNase T2 acts as a tumor suppressor in a variety of cancers, such as colorectal cancer, ovarian tumors, melanoma, and non-Hodgkin's B-cell lymphoma and acute lymphoblastic leukemia (20–23). Hence, the RNase T2 family is receiving an increasing amount of attention due to its key roles in inflammation and cancer (3, 24–28).

## RNase T2 in Inflammation

Innate immunity is the first line of defense against the invasion of pathogenic microorganisms (29). Innate immune cells express a series of pattern recognition receptors (PRRs) to recognize pathogen-associated molecular patterns (PAMPs) derived from pathogens or damaged cells and to distinguish between “self” and “non-self” (30, 31). Exogenous nucleic acids, most commonly present in viral infections, promote the innate immune response by activating corresponding PRRs. RNases can hydrolyze RNA to generate fragments as ligands of PRRs to trigger an immune response. However, various RNases may produce distinct nucleotide motifs that bind to different PRRs due to the localization, cleavage site, RNA specificity, etc. (Table 1).

**Figure 2:**
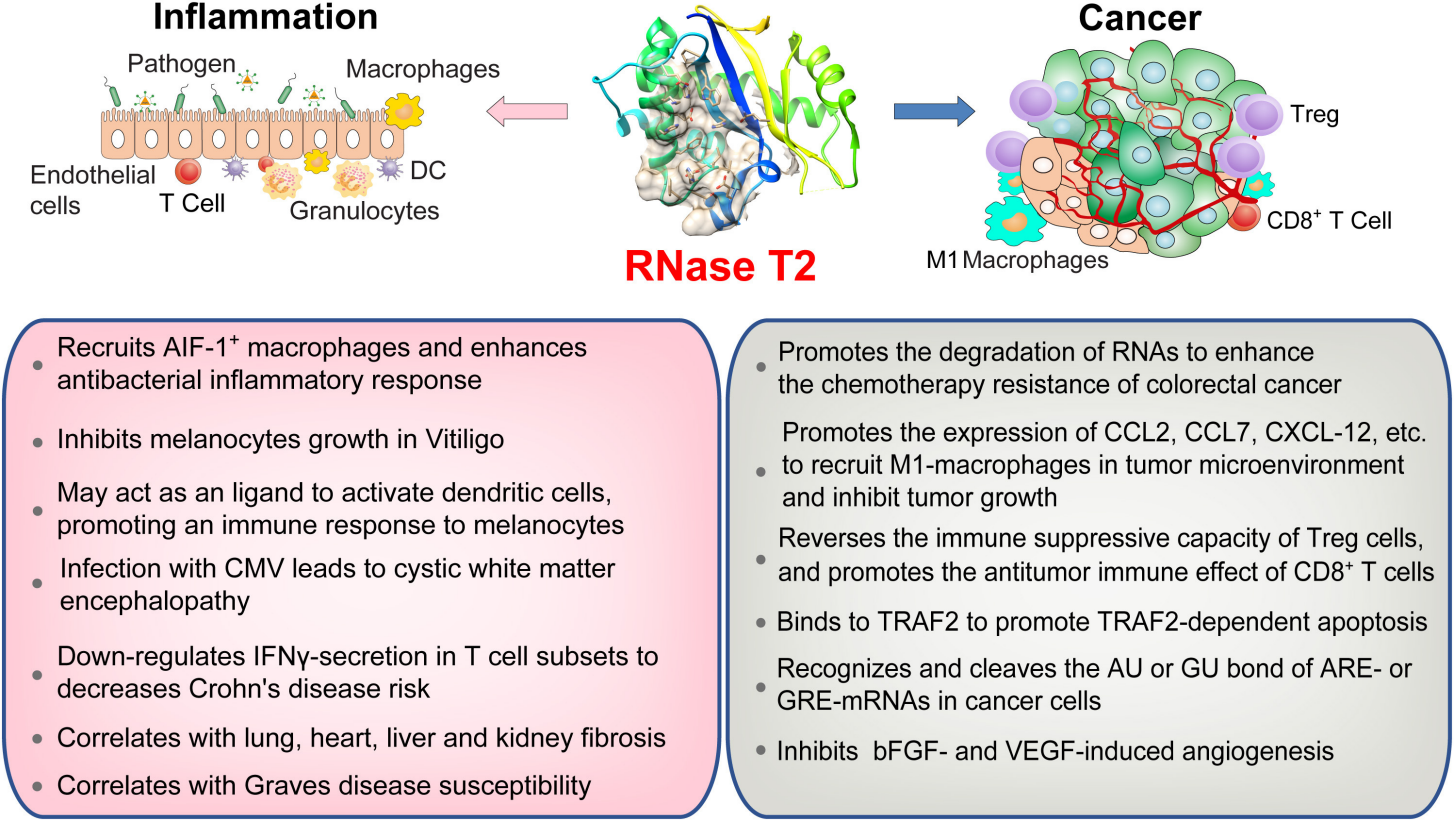
An overview of RNase T2 functions in inflammation and cancer. RNase T2 is critical for defending against the infection of exogenous pathogens. RNase T2 is secreted by granulocytes to recruit macrophages and trigger the innate immune response during pathogen infection. RNase T2 has antitumorigenic activity through promoting cancer cell apoptosis, inhibiting angiogenesis, and enhancing antitumor immunity.

Toll-like receptors (TLRs) are the most studied types of PRRs. To date, four members of the human TLR family can recognize nucleic acids: TLR3, TLR7, TLR8, and TLR9 (53). TLR8 is the most expressed PRR in the human bone marrow cavity and senses RNA from a variety of pathogens, including bacteria and viruses (32). The dimer structure of TLR8 forms two ligand-binding pockets, and TLR8 activation requires both binding pockets to be occupied (54). Previous studies have shown that RNA containing UUGU motifs can cause TLR8-dependent immune responses (55). A recent study published in Cell identified that RNase T2 prefers to cleave between GU or AU bases of ssRNAs to produce two nucleotide fragments with 2'3'-cyclic phosphate adenosine/guanosine terminus and uridine residue, respectively (32). These cleavage products, respectively occupy both binding pockets of TLR8 to activate the anti-pathogen immune response. The deletion of RNase T2 almost completely blocks TLR8 recognition of ssRNA. However, as a control, RNase A cleaved ssRNAs mainly produce pyrimidine-terminated nucleotide fragments, which cannot match the second binding pocket of TLR8. This study provides an interesting peek into the role of RNase T2 in resisting bacterial infections via its immunomodulatory functions (Figure 2) (3, 24).

Similarly, RNase T2 secreted by eggs of *Schistosoma mansoni*, i.e., omega-1, can promote the polarization of CD4^+^ T cells to Th2 through dendritic cells (DCs) (56, 57). It is interesting that omega-1 can change the cytoskeleton structure and function of DCs after being absorbed by DCs. This function seems to be related to its RNase activity because inhibiting its ribonuclease activity can inhibit its role in Th2 polarization (57). In addition, endogenous RNase T2 is either secreted passively by necrotic tissue cells or actively by immune cells or epithelial cells to signal to the innate immune system the occurrence of tissue damage events. More and more studies report that RNase T2 can act as an alarm-like molecule (alarmin), which acts on the innate immune system to send “dangerous” signals (such as bacterial infection, tissue damage, etc.) (58, 59). Earlier studies have shown that RNase T2 is constitutively expressed at low basal levels in healthy animals and that granulocytes produce and secrete RNase T2 after lipopolysaccharide treatment (13, 60). RNase T2 recruits allograft inflammatory factor 1 (AIF-1)-positive macrophages to enhance the antibacterial inflammatory response (13, 61). These studies indicate that RNase T2 has important functions in antibacterial immune processes.

The antiviral activities of RNase T2 have also been reported (3, 62). Elevated levels of extracellular RNase T2 expression resulted in increased resistance to Cucumovirus and Virgaviridae infection in plants (62). In contrast, mutation in RNase T2 is the cause of an autosomal recessive disease of cystic white matter encephalopathy (63). This particular neurological abnormality is related to the loss of RNase T2 in infants before birth. Although it is unclear how RNase T2 deficiency leads to this phenotype, infection with congenital cytomegalovirus (CMV) may be one of the factors (64). Inactivation or loss of RNase T2 due to CMV infection may lead to reduced degradation of extracellular and/or intracellular ssRNAs, which will allow the virus to replicate and trigger the innate immune response and will also affect the infant's nervous system development. Interestingly, to deal with cellular antiviral mechanisms, viruses are able to suppress or inactivate endoribonuclease. Human T-cell Leukemia Virus type 1 (HTLV-1), the pathogen of Adult T-cell leukemia (ATL), encodes a protein Tax and is found to inhibit RNase T2 expression by occupying *RNASET2* gene promoter (65). Similarly, RNase T2 reduction is required for the replication of both the Hepatitis C and dengue viruses (66). Therefore, RNase T2 is regulated upon viral infections and is involved in the modulation of virus reproduction.

Recent studies have linked RNase T2 to susceptibility to autoimmune diseases, such as Crohn's disease (CD), vitiligo, and organ fibrosis. In CD, RNase T2 is the only colitis risk-associated gene that is downregulated more than 5-fold in interferon-γ (IFNγ) -secreting T-cell subsets (16). Decreased RNase T2 expression is closely related to IFNγ production mediated by tumor necrosis factor superfamily member 15 (TNFSF15), which suggests a potential biomarker for patients with severe CD. Restoring the expression of RNase T2 in T cells and reducing the production of IFNγ by T cells benefits the treatment of CD. Vitiligo is an acquired pigmented disease characterized by the loss or destruction of functional melanocytes and depigmented lesions in different parts of the skin (17). The expression level of RNase T2 was enhanced in specimens of patients with vitiligo. The overexpression of RNase T2 can induce the stress response in primary human melanocytes and keratinocytes cultured *in vitro* and inhibit the growth of melanocytes (67). On the other hand, RNase T2 may act as an endogenous ligand to activate antigen-presenting cells, such as DCs, thereby initiating an immune response to melanocytes (68). Organ fibrosis is characterized by fibroblast activation and massive extracellular matrix (ECM) deposition, which can lead to loss of function of the lung, heart, liver, or kidney (69). Similarly, reduced expression of RNase T2 is associated with multiple organ fibrosiss, including lung, heart, liver, and kidney (69).

Genome-wide association studies (GWAS) have boosted our knowledge of genetic risk variants in autoimmune diseases. A GWAS study showed a positive correlation between the *RNASET2* gene rs9355610 SNP locus and Graves' disease (GD) susceptibility in the Chinese Han population (70). Moreover, the G allele of rs9355610 may be a protective factor for liver damage (LD) in patients with GD (71). These data suggest that RNase T2 has a potential intervention effect on GD and LD and provide a new target for the diagnosis and targeted therapy of GD combined with LD. Primary biliary cholangitis (PBC) is an autoimmune liver disease, which is characterized by a chronic cholestasis process that affects the small and medium-sized bile ducts. It is caused by immune-mediated epithelial destruction and causes cholestasis, liver damage, and cirrhosis (72). Recent genetic studies, including twin analysis, family studies, and GWAS, have revealed that RNase T2 (rs9355610) variants correlate with the liver function and metabolic characteristics in patients with PBC (73). Rheumatoid arthritis (RA) is a complex autoimmune disease characterized by chronic inflammation (74). A recent study revealed differences in the racial genetic background of RA susceptibility in European and Asian populations and found a long list of overlapping or race-specific RA-related genes, including RNase T2 (75). This research not only improved our understanding of genetic susceptibility to RA but also provided important insights into the ethnic genetic homogeneity and heterogeneity of RA in different ethnic groups. These observations indicate that RNase T2 is involved in the progression of a variety of autoimmune-related diseases, but how RNase T2 plays a role in these diseases remains to be further studied. With the deepening of GWAS research, further exploration of the role of RNase T2 in regulating immune tolerance and autoimmune-related diseases may help develop new therapeutic targets.

## RNase T2 in the Biology and Immunity of Cancer

**Figure 3:**
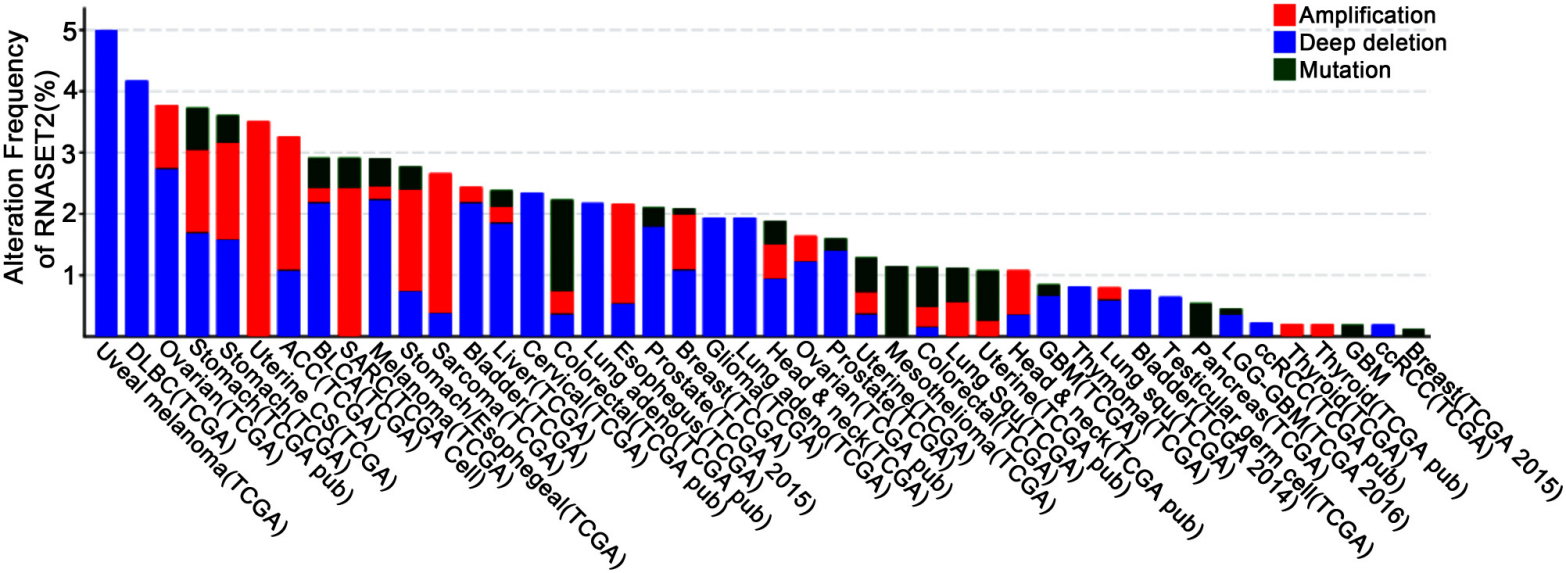
Genetic alterations of *RNASET2* in human cancers. Frequency of *RNASET2* genetic alterations in human tumors from the Cancer Genome Atlas (TCGA) database. Types of alterations include amplification, deep deletion, and mutations.

In addition to the immunomodulatory function in inflammation, RNase T2 is also implicated as a tumor suppressor (5, 18, 19). It has been reported that RNase T2 expression is reduced by 30% in the tumor microenvironment of primary ovarian cancer, and its expression level is also significantly reduced in lymphomas (20, 76). Moreover, the introduction of RNase T2 inhibits the clonogenicity of ovarian cancer cells *in vitro* (20) and suppresses tumorigenesis (20, 77) and metastatic potential (78) *in vivo*. However, the inactivation of RNase T2 through mutation or denaturation still possesses anti-cancer effects, indicating a cleavage-independent role for RNase T2 in tumor suppression (5, 18, 19). Besides, using bioinformatics analysis of The Cancer Genome Atlas (TCGA) database, we found that the expressions and mutations of *RNASET2* in tumors from different tissues are specific (Figure 3). Therefore, the role of RNase T2 in tumor cells may be cancer-type-dependent and location-specific, and its roles in different subcellular locations in tumor cells need to be further studied.

Recent results have indicated that intracellular RNase T2 isoforms can transcriptionally inhibit the expressions of certain mRNA decay-related genes (8, 79, 80). AU-rich Elements (AREs) and GU-rich Elements (GREs) located in the 3'-untranslated region can regulate mRNA stability at the post-transcriptional level (28). The gene encoding products of AREs and GREs, such as *C-MYC*, Epidermal Growth Factor (*EGF*), Prostaglandin-Endoperoxide Synthase 2 (*PTGS2*), Proto-Oncogene C-Jun (*JUN*), and Proto-Oncogene C-Fos (*FOS*), are closely related to tumor processes such as cell growth, apoptosis resistance, angiogenesis, invasion, and metastasis (14, 15). Given that RNase T2 has the function of specifically recognizing and cutting the phosphodiester bond of AU or GU in single-stranded RNA (32), it can be speculated that intracellular RNase T2 may preferentially recognize and cleave the AU or GU bond of AREs or GREs in normal cells, degrading the 3'-terminal poly-A structure, thereby reducing the stability and transcriptional activity of ARE- or GRE-mRNAs (81, 82). Indeed, compared to normal tissues, RNase T2 is significantly reduced in ovarian cancer, lymphoma, chronic lymphocytic leukemia, and melanoma (20), which may result in an increase in ARE- and GRE-genes and thereby promote tumor progression. Nonetheless, future work will be required to determine whether the endogenous RNase T2 also displays tumor suppressive action independent of the ribonuclease activity.

Apoptosis tolerance is an important cause of the failure of chemotherapy in tumors. During 5-Fluorouracil (5-Fu) treatment of colorectal cancer, ABHD5 competitively binds PDIA5, activates RNase T2 in the lysosome, promotes autophagy degradation of RNAs to form uracil, and thus maintains cell survival. The absence or inactivation of RNase T2 can enhance 5-Fu-induced apoptosis of colorectal cancer cells (83). On the other hand, RNase T2 contains PKQE, a potential binding site of tumor necrosis factor receptor-associated factor 2 (TRAF2) (11). It modulates mitogen-activated protein kinase (MAPK) and NF-κB pathways *via* directly binding to TRAF2 (84). During the process of apoptosis induced by tumor necrosis factor, RNase T2 binds to TRAF2 and promotes TRAF2-dependent apoptosis (11). Hence, RNase T2 may play opposing roles in the apoptosis induced in different ways. Moreover, RNase T2 can inhibit angiogenesis induced by angiogenin, basic fibroblast growth factor (*bFGF*), and vascular endothelial growth factor (*VEGF*) in a dose-dependent manner in human umbilical vein endothelial cells and an LS174T-derived xenograft mouse model (77). Consistently, the human RNase T2-derived peptide trT2-50 inhibits angiogenesis, clonal colony formation, and tumor progression *in vitro* (85).

The alterations in the tumor immune microenvironment are also important factors that determine the ultimate fate of the tumor. RNase T2 can promote the expression of monocyte/macrophage chemokines, including C-C motif chemokine ligand 2 (CCL2), CCL7, and C-X-C motif chemokine ligand (CXCL)-12. RNase T2 *per se* can directly bind and recruit monocytes and macrophages (86). Exogenous RNase T2 promotes the infiltration of M1-type macrophages into the tumor microenvironment to inhibit tumor growth (5, 86). In addition, Poly-G3 (ssRNA40) can activate TLR8 to cause regulatory T-cell (Treg) metabolism reprogramming, inhibit glucose absorption and glycolysis, reverse the immune-suppressive capacity of Treg cells, and promote the antitumor immune effect of CD8^+^ T cells (87). Since RNase T2 shear processing is a prerequisite for TLR8 to recognize ssRNA40, RNase T2 may change the tumor microenvironment by regulating the number and function of Treg and CD8^+^ T cells. These results indicate that RNase T2 participates in tumor immunity and suggest a potential strategy for cancer immunotherapy.

## Potential Applications of RNase T2

In view of the above studies, RNase T2 has broad application prospects in antibacterial infection and antitumor treatment (25). Some functional small fragments of RNase T2 can be used as biological agents that provide anti-infective and antitumor effects. Special receptors may distinguish RNAs produced by exogenous cells and their own RNAs. On the one hand, recognition of exogenous RNAs will activate the appropriate immune response to clear invaders (88). On the other hand, some exogenous RNA appears in places where it is impossible to generate RNA of its own origin, such as lysosomes engulfed by cells. Cells can also adjust the threshold for foreign nucleic acid recognition through other secondary signals such as interferon, indicating that different types and parts of RNA may be sensed and recognized by different receptors.

The RNase T2 family has been well-characterized to selectively cleave tRNA or rRNA under oxidative stress (80, 89, 90), and specific tRNA-derived fragments (tRFs) were found to be associated with ARGONAUTE (AGO) proteins in human and plants (90). An attractive idea is that RNaseT2 can control the activity of small RNAs (91, 92). Nowadays, small RNA-mediated gene silencing technology has become a weapon in gene function research and has been widely used. Similar to CRISPR/CAS-9 (93), whether RNase T2, a conserved endonucleases that drives an innate immune defense against foreign RNA invasion, can be used for RNA editing in organisms (viruses use RNA as their genetic material) requires further exploration. Moreover, during the RNA interference process, RNase T2 can cleave exogenous RNA at the G and U residue positions (26), but what proteins are included in this cleavage complex? Is the cleavage of the target site guided by small RNA molecules and involved in recognition (94, 95)? These questions should be addressed before the application of RNase T2 in gene editing.

SARS-CoV-2 (also named 2019-nCoV), a novel ssRNA coronavirus, is causing an outbreak of unusual viral pneumonia in patients and is spreading worldwide. Recently, its sequence has been deciphered (GeneBank No. MN908947) (96). As previously mentioned, several viruses are able to suppress RNase T2 expression to facilitate their reproduction (65), and it seems likely that RNase T2 acts as a barrier for virus penetration. Thus, we speculate that SARS-CoV-2 might play a similar role during the infection process, and RNase T2 might be suppressed during infection with SARS-CoV-2. However, whether the level of RNase T2 is changed during infection with SARS-CoV-2 remains to be evaluated, whether it can be used to cleave this virus to attenuate coronavirus-related pneumonia is unknown. Further investigations of the functional role of RNase T2 in SARS-CoV-2 infection are imperative before this endonuclease can be applied as a potential anti-viral agent.

## Concluding Remarks

In conclusion, RNase T2 has been suggested to participate in many biological processes, including inflammation and cancer, via ribonuclease-dependent and independent mechanisms. However, RNase T2 has different functions in different locations, which makes it difficult to study the role of RNase T2 in immune regulation. In future studies, scientists need to (1) analyze the specific coding information or post-translational modification of RNase T2 subtypes in different subcellular locations, (2) explore by which cells RNase T2 are secreted in the immune microenvironment, (3) find out what kind of receptors the secreted RNase T2 binds and how innate immunity is activated, (4) elucidate whether the endonuclease activity of exogenous RNase T2 has any effect on immunity, and (5) verify whether intracellular RNase T2 can regulate the stability and transcriptional activity of ARE- and GRE-containing mRNAs. Given that RNase T2 plays a key role in resisting infectious verification and non-infectious inflammation and enhancing tumor immunity, further observation of the regulatory mechanism of RNase T2 in inflammation and tumor immunity will be useful for future clinical studies on the initiation and regression of inflammation and the pathological mechanisms of tumorigenesis and progression. This will provide an important theoretical and experimental basis for the development of new methods for diagnosis and treatment of related diseases.

## Author Contributions

YL conceived and supervised the manuscript. LW, HZ, and YL contributed to the preparation of figures. LW, YX, HZ, and YL wrote and revised the manuscript. All authors contributed to the article and approved the submitted version.

## Conflict of Interest

The authors declare that the research was conducted in the absence of any commercial or financial relationships that could be construed as a potential conflict of interest.
